# A bibliometric and visual analysis of obesity and polycystic ovary syndrome from 2012 to 2022

**DOI:** 10.3389/fendo.2022.1011105

**Published:** 2022-11-02

**Authors:** Ping Luo, Jiake Li, Pengzhou Li, Guohui Wang, Weizheng Li, Zhi Song, Xulong Sun, Zhibing Fu, Hui Zhou, Xianhao Yi, Liyong Zhu, Shaihong Zhu

**Affiliations:** Department of General Surgery, Third Xiangya Hospital, Central South University, Changsha, Hunan, China

**Keywords:** obesity, polycystic ovary syndrome, bibliometric analysis, visualization, VOSviewer

## Abstract

**Background:**

Obesity is associated with polycystic ovary syndrome (PCOS). We aimed to elucidate the research status and explore research trends and future directions of research on obesity and PCOS.

**Methods:**

A bibliometric analysis of the published papers in the field of obesity and PCOS between 2012 and 2022 was conducted on the basis of the Web of Science Core Collection database. The collaboration networks, research trends, literature sources, citation analysis, co-citation analysis, and keywords analysis were statistically analyzed and visualized using the VOSviewer software.

**Results:**

We retrieved 2843 records from 681 journals by 12307 authors from 2942 institutes in 99 countries. The number of published papers and citations had a roughly increasing trend annually. The United States and China contributed the majority of the records. Monash University, Shanghai Jiaotong University, Aristotle University of Thessaloniki, Karolinska Institute, University of São Paulo, and Tehran University of Medical Sciences were the biggest nodes in their cluster of the collaboration network map, and Moran LJ, Teede HJ, Joham AE, Escobar-Morreale HF, and Macut D were prolific authors. Research trends and hotspots were identified and visualized in the field of obesity and PCOS. Research hotspots in this field focused on insulin resistance (IR), metabolic syndrome, metformin, and inflammation. Bariatric surgery, mitochondrial dysfunction, binding globulins, and comorbidities may be the frontiers of future research.

**Conclusions:**

We concluded the research status and trends in the field of obesity and PCOS. A better understanding of collaboration patterns, research hotspots, and frontiers may be useful for researchers.

## 1 Introduction

Obesity, defined as abnormal or excessive fat accumulation that may impair health, is a worldwide pandemic disease, especially in women (1, 2). Obesity increases the risk of multiple diseases, such as diabetes mellitus (3), cardiovascular disease (3, 4), and PCOS (5). PCOS, characterized by ovulatory dysfunction, hyperandrogenism, and polycystic ovaries, is one of the most common heterogeneous endocrine conditions and affected 5% to 10% of reproductive-age women, depending on the Rotterdam criteria (6, 7). Approximately 50% of women with PCOS are overweight or obese (8). Although the etiology of PCOS remains unclear, IR and hyperandrogenemia (HA) play a major role in the pathogenesis of PCOS development and complications (9). However, obesity is strongly associated with IR and HA. Obesity amplifies and worsens the adverse metabolic and reproductive outcomes of PCOS and increases IR and compensatory hyperinsulinemia and generates testosterone while suppressing gonadotropin production (10). Meanwhile, Obesity sensitizes thecal cells to luteinizing hormone stimulation and amplifies HA by upregulating ovarian androgen levels (11). The symptoms of PCOS can be improved with weight loss through lifestyle modification, excise, drug therapy, and bariatric surgery (12). However, the pathophysiologic mechanism and intricate relationship between obesity and PCOS remain uncertain, numerous studies have been conducted to identify the question (13–15).

Based on this, it is necessary for us to learn about the advances and novel trends in this field. Bibliometric analysis, an approach to quantifying and visualizing published documents, can provide an overview of the current research status and establish future research orientations in a specialized field (16). Bibliometric analysis revealed the research characteristics, status, and trends of PCOS in specific times and regions (17, 18). Some studies also have explored the research status, hotspots, and trends of research between PCOS and infertility as well as IR by the method of bibliometrics (19, 20). To the best of our knowledge, no bibliometric analysis has focused on the collaboration patterns and research trends, and hotspots of research on obesity and PCOS. To clarify the current research status and provide references for future research, we performed a bibliometric analysis to quantify and visualize this research field through analysis of the information in published papers.

## 2 Materials and methods

### 2.1 Literature source and retrieval

The retrieved documents were based on the Web of Science Core Collection (Science Citation Index Expanded, SCI-E) from 1 January 2012 to 27 July 2022. The detailed retrieval strategy was as follows: TS= (“Polycystic Ovary Syndrome” OR “Polycystic Ovarian Syndrome” OR “Stein-Leventhal Syndrome”) AND TS= (“obesity”). The document type was limited to articles and reviews. Only publications in English were included.

### 2.2 Inclusion and exclusion criteria

Inclusion criteria were peer-reviewed published original articles or reviews about obesity and PCOS. Exclusion criteria were: 1) Early access, meeting abstract, editorial material, and other types; 2) repeated publications.

### 2.3 Literature analysis methods

All the records were downloaded as plain text and imported to VOSviewer to conduct bibliometric analysis. Corresponding data statistics and analysis were used by Excel 2019 (Microsoft) and GraphPad Prism (version 8.0). VOSviewer (version 1.6.18, Leiden University) was an available software for creating and viewing bibliometric maps and was used for visualizing the collaborations between countries, institutions, authors, keywords, and references (21). Each node represents an individual, the same color represents the same cluster, the size of the circles means the number or frequency of individuals, and the lines between circles express the intensity of cooperation (22).

## 3 Results

### 3.1 Search results

**Figure 1:**
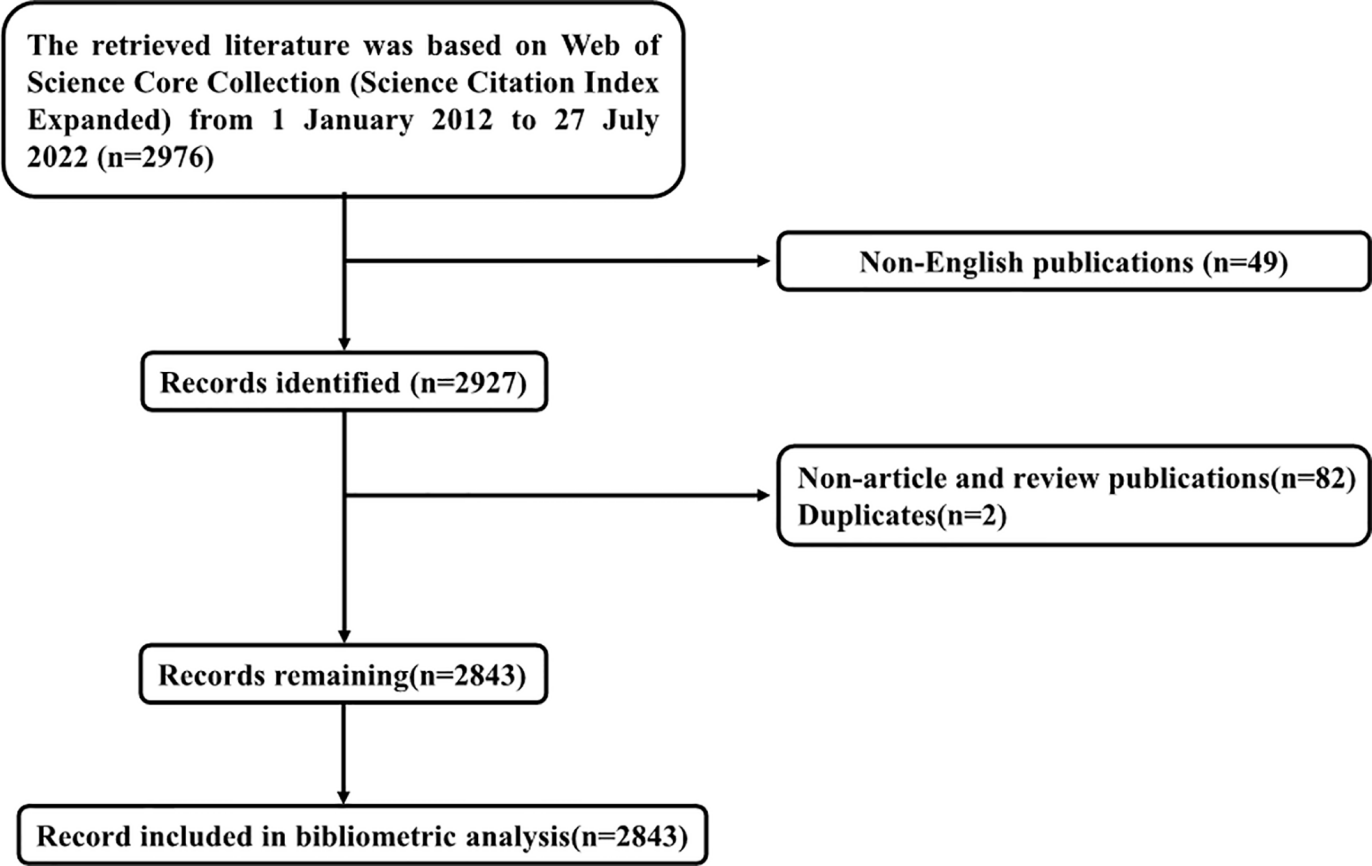
Flow chart of document selection and identification.

Totally 2976 publications were identified from SCI-E. Duplicates, non-article and review publications, and non-English publications were excluded. Finally, 2843 was identified as the number of published records and included in the final bibliometric analysis (**Figure 1**).

### 3.2 General trends and annual publications

**Figure 2:**
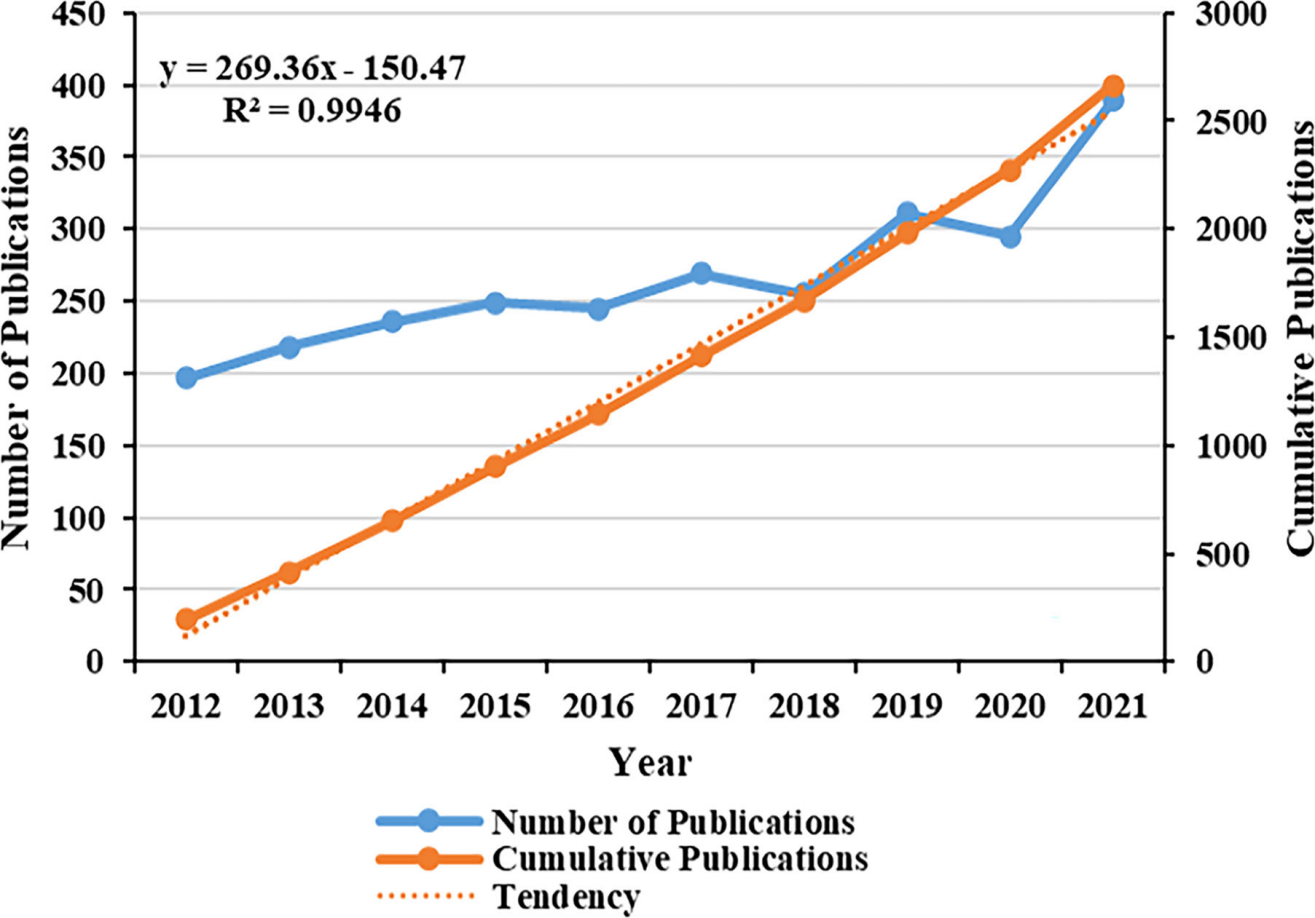
Annual and cumulative publications.

**Figure 3:**
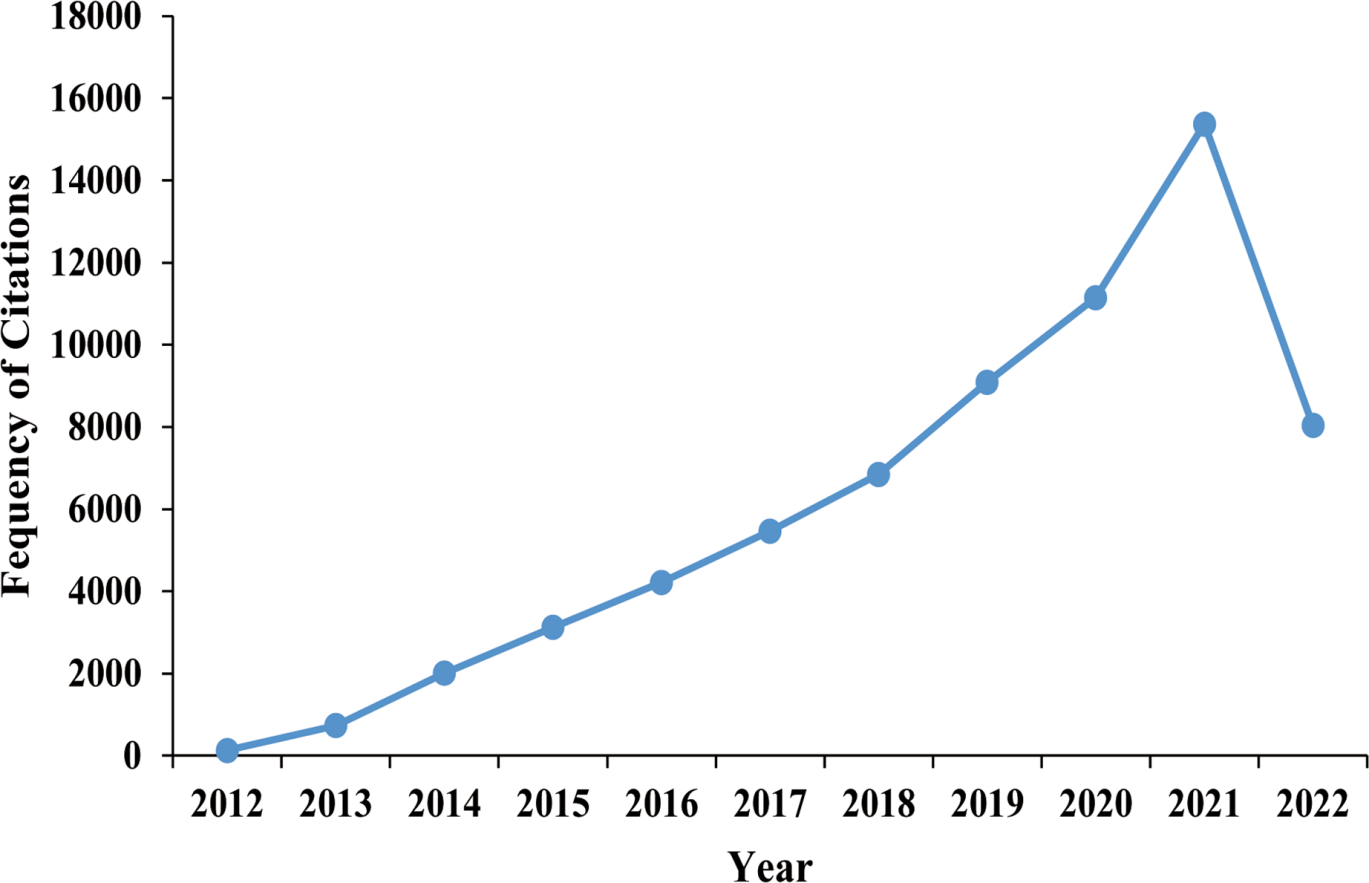
Annual frequency of citations.

Papers, published over the past 10 years, roughly conformed to the increasing trend year by year, and the cumulative number of published articles showed an overall upward trend (**Figure 2**). Most records were original articles and reviews (2843/2927, 97.1%), which can greatly reflect the development trends and changes in the field of obesity and PCOS. Through database searching, the 2843 documents were cited 66178 times, with an average of 23.3 times per paper, and an H-index of 101 (**Figure 3**). It can be seen that the direction of obesity and PCOS has attracted more and more researchers’ attention.

### 3.3 Countries and districts

Ninety-nine countries contributed to publications in the field of obesity and PCOS worldwide. The top 3 countries that published papers were the United States, China, and Turkey, accounting for 47.7% (1356/2843). Australia had the highest average number of citations (38.9), followed by the United Kingdom (38.4), the United States (37.2), Italy (35.6), and Turkey (15.4) (**Table 1**).

**Table 1:** Analysis of top 10 productive countries.

Rank	Country	Documents, n	Citations, n	Average citations	Total link strength
1	United States	636	23718	37.2	279
2	China	508	6595	12.9	128
3	Turkey	212	3267	15.4	70
4	United Kingdom	198	7621	38.4	214
5	Italy	163	5803	35.6	131
6	Australia	161	6273	38.9	126
7	India	123	1771	14.3	41
8	Poland	122	1549	12.6	35
9	Iran	120	1579	13.1	29
10	Brazil	116	1783	15.3	37

**Figure 4:**
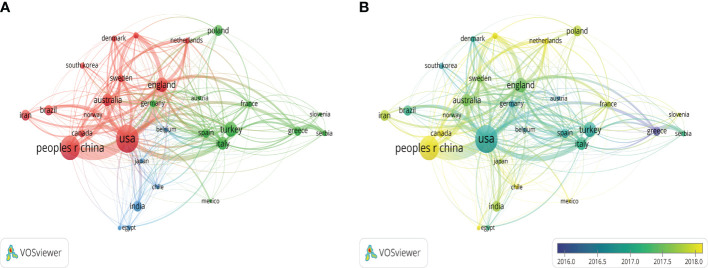
Visualization map of countries and districts. **(A)** Network diagram of the top 30 countries/districts. **(B)** Dynamics and trends of the top 30 countries/districts.

The top 30 most prolific countries had formed 3 stable clusters and created a network map. There was active cooperation between these countries, especially between the United States and China (**Figure 4A**). Viewed from the dynamics and trends, research about obesity and PCOS mainly focused on the United States, Greece, Australia, and Turkey from 2012 to 2017. The field and direction gradually received greater attention from other countries after 2017 (**Figure 4B**).

### 3.4 Universities and institutions

**Figure 5:**
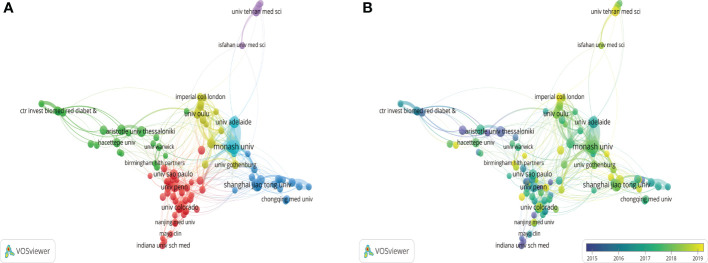
Visualization map of universities and institutions. **(A)** Network diagram of the top 100 universities/institutions. **(B)** Dynamics and trends of the top 100 universities/institutions.

A total of 2942 institutions were involved in these documents. Collaborative network analyses have been conducted between universities or institutions. In a network and overlay visualization of institutions in the top 100, 6 clusters were formed and the biggest node represented that the institution published the most article in their cluster (**Figure 5A**). Monash University (n=88, 3.1%, Cluster 6), Shanghai Jiaotong University (n=57, 2.0%, Cluster 3), Aristotle University of Thessaloniki (n=41, 1.4%, Cluster 2), Karolinska Institute (n=40, 1.4%, Cluster 4), University of São Paulo (n=38, 1.3%, Cluster 1), and Tehran University of Medical Sciences (n=32, 1.1%, Cluster 5) were the biggest nodes in their cluster, Cluster 1 was the biggest cluster, which contained 40 nodes that represented its related different universities or institutions, while Cluster 6 was the smallest, which included 4 nodes. The other clusters included 20 nodes (Cluster 2), 19 nodes (Cluster 3), and 12 nodes (Cluster 4). Furthermore, the results showed that 3 universities mainly contributed to this field from 2012 to 2017, which included Aristotle University of Thessaloniki, The University of Adelaide, and University of São Paulo. Since then, these studies have gradually gained popularity among other institutes, such as Monash University, and Shanghai Jiaotong University (**Figure 5B**).

### 3.5 Authors

**Figure 6:**
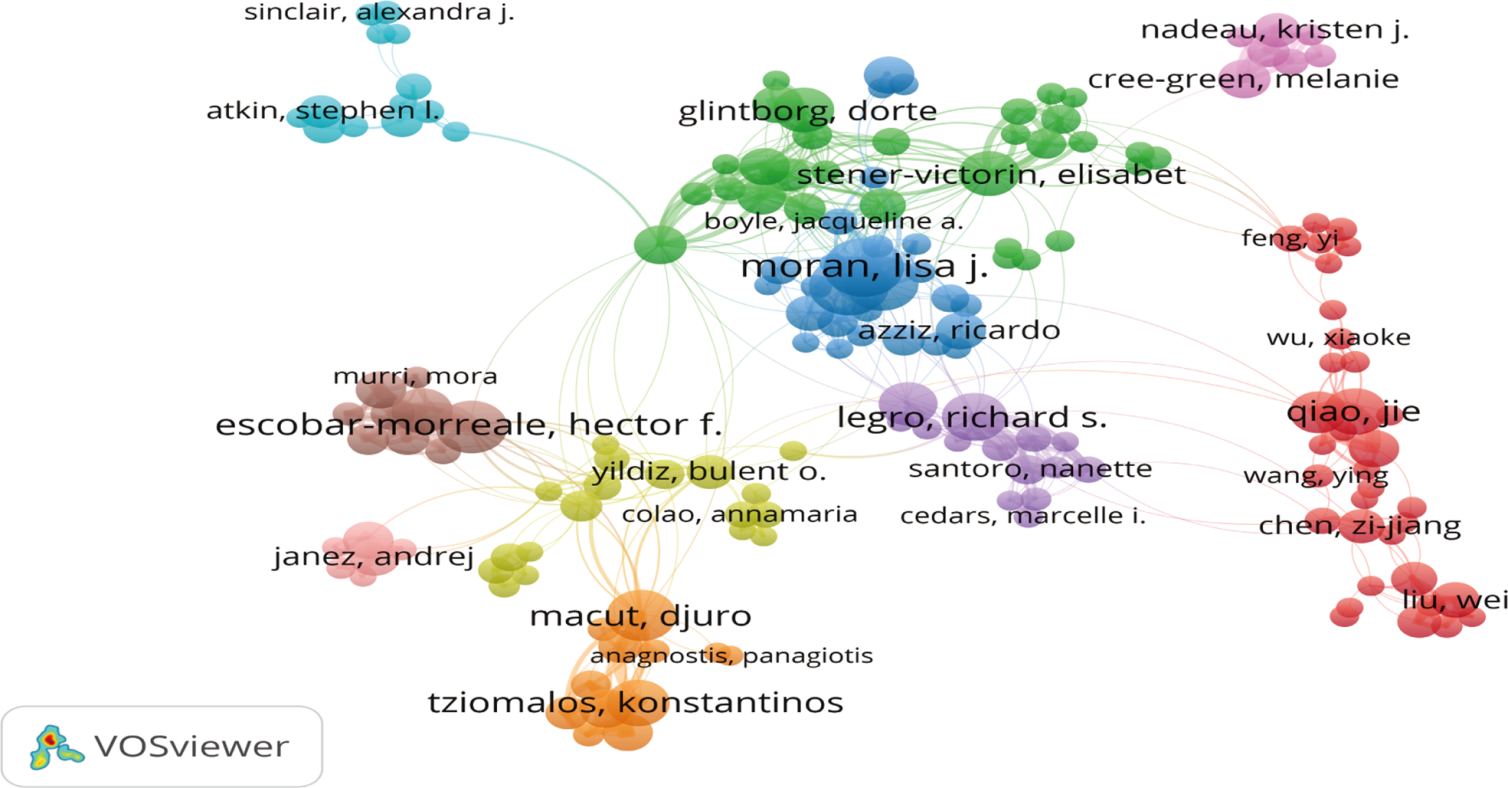
Network map of the top 150 authors.

Totally 12307 authors contributed to the publications in this domain. The author visualization map of the top 150 productive authors formed 10 clusters (**Figure 6**). The biggest cluster was Qiao Jie (34/12307), who came from Peking University. Stener-Victorin E (30/12307) had the second biggest cluster and was from Karolinska Institute. The third biggest cluster was Moran LJ (27/12307) who has published the most documents and collaborated with other researchers frequently (**Figure 6** and **Table 2**).

**Table 2:** Analysis of top 10 productive authors.

Rank	Author	Documents, n	Citations, n	Average citations	Total link strength
1	Moran LJ	36	1268	35.2	91
2	Teede HJ	35	1360	38.8	93
3	Joham AE	31	840	27.0	94
4	Escobar-Morreale HF	30	2123	70.7	100
5	Macut D	27	892	33.0	71
6	Legro RS	25	1183	47.3	58
7	Tziomalos K	23	493	21.4	71
8	Qiao J	23	962	41.8	35
9	Luque-Ramirez M	22	962	43.7	79
10	Stener-Victorin E	22	1130	51.3	71

### 3.6 Journals

This study showed that a total of 681 journals published records about obesity and PCOS. The results showed that the most productive journal was Gynecological Endocrinology (n=156), the most cited journal was The Journal of Clinical Endocrinology & Metabolism (4100 citation times), and the journal with the highest average citation was Endocrine Reviews (n=6, an average citation was 298.1 times). The top productive journals, top cited journals, and journals of top average citations were listed (n=10) in **Supplement File 1**.

### 3.7 Citations and co-citations

#### 3.7.1 Top 10 citation publications

Of the 2843 records, the top 10 publications ranked by citation were listed in **Table 3**. The most cited article was published in Endocrine Reviews by Diamanti-Kandarakis E et al. in 2012 (23) and reported an update on mechanisms and implications of IR and PCOS with 874 citations, which was higher than that of the second paper (525 citations) (24). The third cited paper was on the pathogenesis of PCOS, with 454 citations (10).

**Table 3:** Analysis of top 10 citations.

Rank	Author	Number of citations	Title	Journal
1	Diamanti-Kandarakis E, 2012	874	Insulin resistance and the polycystic ovary syndrome revisited: an update on mechanisms and implications	Endocrine Reviews
2	Manikkam M, 2013	525	Plastics derived endocrine disruptors (BPA, DEHP and DBP) induce epigenetic transgenerational inheritance of obesity, reproductive disease and sperm epimutations	PloS One
3	Rosenfield RL, 2016	454	The Pathogenesis of Polycystic Ovary Syndrome (PCOS): the Hypothesis of PCOS as Functional Ovarian Hyperandrogenism Revisited	Endocrine Reviews
4	Escobar-Morreale HF, 2018	449	Polycystic ovary syndrome: definition, aetiology, diagnosis and treatment	Nature Reviews Endocrinology
5	Dumesic DA, 2015	390	Scientific Statement on the Diagnostic Criteria, Epidemiology, Pathophysiology, and Molecular Genetics of Polycystic Ovary Syndrome	Endocrine Reviews
6	Lim SS, 2012	386	Overweight, obesity and central obesity with polycystic ovary syndrome: a systematic review and meta-analysis	Human Reproduction Update
7	Garvey WT, 2016	349	American association of clinical endocrinologists and American college of endocrinology comprehensive clinical practice guidelines for medical care of patients with obesity	Endocrine Practice
8	Yildiz BO, 2012	347	Prevalence, phenotype and cardiometabolic risk of polycystic ovary syndrome under different diagnostic criteria	Human Reproduction
9	Conway G, 2014	335	The polycystic ovary syndrome: a position statement from the European Society of Endocrinology	European Journal of Endocrinology
10	Murri M, 2013	273	Circulating markers of oxidative stress and polycystic ovary syndrome (PCOS): a systematic review and meta-analysis	Human Reproduction Update

#### 3.7.2 Co-citation references

**Figure 7:**
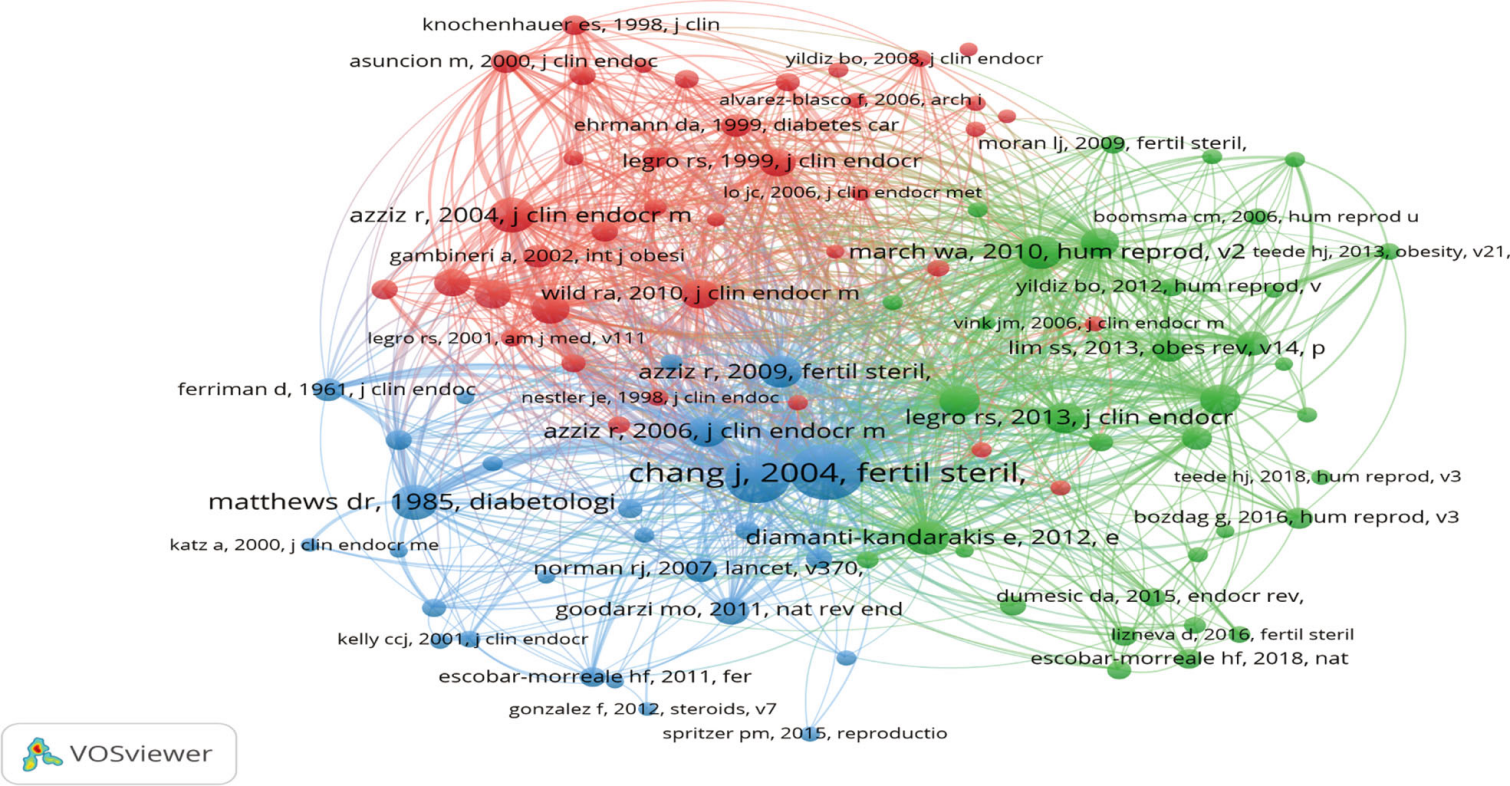
Network map of co-citation references.

VOSviewer was used to visualize the co-citation network of references, which were divided into 3 clusters, with 93571 references (**Figure 7**). The larger node represents that the references are cited more frequently, and the line thickness represents the co-citation frequency. The top 10 co-cited references were shown in **Table 4**. And these co-citation papers were mainly published in Fertility and Sterility, The Journal of Clinical Endocrinology & Metabolism, and Human Reproduction.

**Table 4:** Analysis of top 10 co-citations.

Rank	Author	Number of citations	Title	Journal
1	Chang J, 2004	679	Revised 2003 consensus on diagnostic criteria and long-term health risks related to polycystic ovary syndrome	Fertility and Sterility
2	Fauser BCJM, 2004	517	Revised 2003 consensus on diagnostic criteria and long-term health risks related to polycystic ovary syndrome (PCOS)	Human Reproduction
3	Matthews DR, 1985	302	Homeostasis model assessment: insulin resistance and beta-cell function from fasting plasma glucose and insulin concentrations in man	Diabetologia
4	Azziz R, 2004	286	The prevalence and features of the polycystic ovary syndrome in an unselected population	The Journal of Clinical Endocrinology & Metabolism
5	March WA, 2010	278	The prevalence of polycystic ovary syndrome in a community sample assessed under contrasting diagnostic criteria	Human Reproduction
6	Diamanti-Kandarakis E, 2012	264	Insulin resistance and the polycystic ovary syndrome revisited: an update on mechanisms and implications	Endocrine Reviews
7	Azziz R, 2009	253	The Androgen Excess and PCOS Society criteria for the polycystic ovary syndrome: the complete task force report	Fertility and Sterility
8	Azziz R, 2006	229	Positions statement: criteria for defining polycystic ovary syndrome as a predominantly hyperandrogenic syndrome: an Androgen Excess Society guideline	The Journal of Clinical Endocrinology & Metabolism
9	Fauser BCJM, 2012	225	Consensus on women’s health aspects of polycystic ovary syndrome (PCOS): the Amsterdam ESHRE/ASRM-Sponsored 3rd PCOS Consensus Workshop Group	Fertility and Sterility
10	Legro RS 2013	219	Diagnosis and treatment of polycystic ovary syndrome: an Endocrine Society clinical practice guideline	The Journal of Clinical Endocrinology & Metabolism

### 3.8 Analysis of keywords

**Figure 8:**
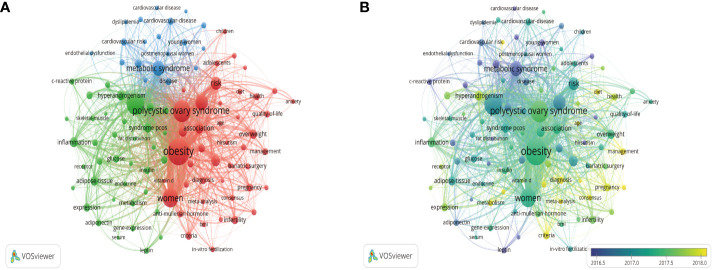
Visualization map of keywords. **(A)** Network diagram of the top 100 keywords. **(B)** Dynamics and trends of the top 100 keywords.

A total of 7462 keywords were extracted from the retrieved records. The network map of the top 100 keywords was grouped into 3 clusters (**Figure 8A**), and most of the top 10 keywords were clustered in the red cluster. The top 3 keywords were “obesity (n=1627)”, “polycystic ovary syndrome (n=1064)”, and “insulin-resistance (n=1037)”. From **Figure 8B** and **Table 5**, we can find that the hot research directions of obesity and PCOS in recent 10 years were insulin resistance, metabolic syndrome, metformin, inflammation, and so on.

**Table 5:** Occurrences and total link strength of top 10 keywords.

Rank	Keyword	Occurrences	Total link strength
1	Obesity	1627	9528
2	Polycystic ovary syndrome	1064	6459
3	Insulin-resistance	1037	6298
4	Women	821	4864
5	Prevalence	533	3486
6	Metabolic syndrome	525	3397
7	Risk	376	2308
8	Body-mass index	282	1558
9	Association	257	1550
10	Metformin	230	1430

### 3.9 Research frontier analysis

By analyzing the keywords from 2021 to 2022, a keyword table of research frontiers was obtained (**Table 6**). According to the table, comorbidities of obesity and PCOS, mitochondrial dysfunction, binding globulin, and bariatric surgery may become the research frontier in the future.

**Table 6:** Analysis of keywords from 2021 to 2022.

Rank	Year	Frequency	Keyword
1	2021	4	Mitochondrial dysfunction
2	2021	3	Metabolic profile
3	2021	3	Sleeve gastrectomy
4	2022	3	Hypertensive disorder
5	2022	2	Fatty liver
6	2022	2	Chronic kidney disease
7	2022	2	Bifidobacterium
8	2022	2	Binding globulin
9	2022	2	Activated protein kinase
10	2022	2	Androgen generation

## 4 Discussion

In contrast to traditional literature reviews, bibliometrics analysis can systematically analyze the published literature in a specific field (25). This study summarizes collaboration networks, research trends and hotspots, and future directions in obesity and PCOS based on bibliometric analysis. A total of 2843 records were retrieved and published in 681 journals by 12307 authors from 2942 institutes in 99 countries.

Based on our results, the number of published articles showed an increasing trend, especially in 2021, which indicated that this field had been a research hotspot, suggesting that novel researchers can engage in this research field. There is a correlation between academic productivity and socio-economic status for most countries (26). Ilagan-Vega et al. revealed that the research and development expenditures were significantly related to the academic impact of PCOS in Southeast Asia (17). In terms of article production, the United States ranked first, followed by China, Turkey, the United Kingdom, and Italy. Most of the top 5 countries with the most articles published were developed countries. The citation rate is an important index to measure the academic influence of a paper, which reflects the acceptance and recognition of a country’s scientific research achievements by other countries or institutions (27). The United States (n=23718) and the United Kingdom (n=7621) were the top 2 countries with the highest total citation frequency, but the average citation frequency in Australia was higher than these two countries, which indicated that Australia had greater influence in this field. It has been reported that international collaboration is a powerful factor in promoting academic productivity in a given field (28). In our analysis, different countries and regions had different degrees of cooperation, the United States, which published the most, showed the strongest links with China, followed by the United Kingdom. At the same time, these countries were also among the top 5 most prolific countries. Regional imbalances were observed in different districts and countries, and it was essential to strengthen collaboration and communication among developed and developing countries worldwide.

Meanwhile, the analysis of institutions and authors was similar to the distribution of countries and districts. Among 2942 institutions, Monash University and The University of Adelaide (Australia), Shanghai Jiao Tong University (China), Karolinska Institute, and Oulu University (European) made great contributions to this field and maintained a steady collaboration with other teams. In the last decade, Moran LJ, published the most articles, mainly focused on lifestyle intervention and metabolomics research in this direction (29–31). Qiao Jie, from Peking University, was in the biggest cluster and mainly focused on the pathogenesis and theoretical research in this area, such as immunity (32), gut microbiota (33), and single-cell sequencing (34).

Obesity and PCOS often interacted with each other and caused a significant risk to women’s health (5). Therefore, it received the attention and recognition of many journal editors. According to our results, hundreds of journals have published articles on this topic. Of 681 journals, the top 10 journals contributed the most. The most published journal was Gynecological Endocrinology, and the most cited journal was The Journal of Clinical Endocrinology & Metabolism, while Endocrine Reviews with the highest average paper citation. In addition, the top 3 most average cited journals in the included records were published in top-ranked journals, which indicated that these journals were widely recognized and deserved the attention of researchers in the field. These top-cited records, published in Endocrine Reviews, PloS One, Human Reproduction Update, and European Journal of Endocrinology, together had been cited more than 1853 times in the past 10 years. The common themes in this field’s top 10 cited research articles were the pathogenesis, epidemiology, diagnosis, and treatment of PCOS and its relationship to obesity. Co-citation analysis of references can tell researchers which references had made important contributions in this field. In co-citation analysis, “insulin resistance”, “mechanisms”, “implications”, and “criteria” were the keywords of the top co-citation references, which deserved reference for new researchers (23, 35, 36).

High-frequency keywords are usually used to identify research hotspots in a research field. Shi et al. revealed that the research trends of PCOS were gradually shifting from treatment to mechanistic exploration (18). According to the results of the keywords network map, we found that the research about obesity and PCOS mainly focused on insulin resistance, metabolic syndrome, drug therapy, and inflammation in recent years, which involved the mechanism, comorbidities, and treatment (37–39). It indicated that, in addition to mechanistic exploration, treatment was still a hot research topic in the field of obesity and PCOS.

The co-occurrence keyword analysis of the past 2 years revealed mechanisms, comorbidities, drug therapy, and bariatric surgery as emerging topics. Among obese girls with PCOS, mitochondrial acylcarnitine (C4) was associated with valine and free fatty acids (40). According to Fatemeh et al. (41), PCOS may reduce the occurrence of silent coronary artery disease in a population-based cohort study. Compared with metformin, bariatric surgery should be prioritized for patients with obesity and PCOS in a Chinese prospective nonrandomized trial (42). In these studies, researchers were able to identify the research hotspots, which may shape future research directions.

## 5 Limitations

Without a doubt, the study had several limitations. Firstly, we only analyzed records from the Web of Science Core Collection and only included English articles and reviews, which might lead to selection bias. However, the number of retrieved papers was large enough to reflect the real research situation (43). Secondly, due to the diversity of keywords and the authors with the same name, it was hard to accurately retrieve all the records.

## 6 Conclusions

Utilizing the retrieved records about obesity and PCOS, we performed a bibliometric and visual analysis. The number of papers published had roughly risen and extensive cooperation was observed between different countries and institutions. In this field, comorbidities, mitochondrial dysfunction, binding globulin, and bariatric surgery were the frontiers of research. Bibliometric analysis of the literature in this field was helpful for researchers to understand the collaboration patterns, research hotspots, and frontiers.

## Data availability statement

The datasets presented in this study can be found in online repositories. The names of the repository/repositories and accession number(s) can be found in the article/**Supplementary Material**.

## Author contributions

PL and JKL designed the study. PZL, GHW, SZ, WZL, ZW, XLS, ZBF, XHY, LYZ, and SHZ searched and analyzed the data. All the authors contributed to writing and approving the final manuscript.

## Funding

This work was supported by the Wisdom Accumulation and Talent Cultivation Project of the Third Xiangya Hospital of Central South University (YX202102).

## Acknowledgments

We thank professor Mingyi Zhao for Providing the methodology.

## Conflict of interest

The authors declare that the research was conducted in the absence of any commercial or financial relationships that could be construed as a potential conflict of interest.

## Publisher’s note

All claims expressed in this article are solely those of the authors and do not necessarily represent those of their affiliated organizations, or those of the publisher, the editors and the reviewers. Any product that may be evaluated in this article, or claim that may be made by its manufacturer, is not guaranteed or endorsed by the publisher.
