# Supplementary material for: A bibliometric and visual analysis of obesity and polycystic ovary syndrome from 2012 to 2022
Source: Front Endocrinol (Lausanne). 2022 Nov 2;13:1011105. doi: 10.3389/fendo.2022.1011105 (PMC9666686; doi:10.3389/fendo.2022.1011105)
Supplement: Supplementary file 1 [file Table_1.docx]

**Supplement file 1** Analysis of top journals and their citations

| **Rank** | **Top productive journals** | | |  | **Top cited journals** | | |  | **Top average citations** | | |
| --- | --- | --- | --- | --- | --- | --- | --- | --- | --- | --- | --- |
|  | **Journals (IF&Q)** | **Total number**  **(n)** | **Percentage (%)** |  | **Journals**  **(IF&Q)** | **Total number**  **(n)** | **Total Citations** |  | **Journals**  **(IF&Q)** | **Total number**  **(n)** | **Average times** |
| 1 | Gynecological Endocrinology (2.2, Q3) | 156 | 5.4% |  | The Journal of Clinical Endocrinology & Metabolism  (6.1, Q1) | 101 | 4100 |  | Endocrine Reviews  (25.2, Q1) | 6 | 298.1 |
| 2 | The Journal of Clinical Endocrinology & Metabolism  (6.1, Q1) | 101 | 3.5% |  | Human Reproduction  (6.3, Q1) | 77 | 2808 |  | Nature Reviews Endocrinology  (47.5, Q1) | 6 | 150.5 |
| 3 | Human Reproduction  (6.3, Q1) | 77 | 2.7% |  | Human Reproduction Update  (17.1, Q1) | 23 | 2443 |  | Human Reproduction Update  (17.1, Q1) | 23 | 106.2 |
| 4 | Fertility and Sterility  (7.4, Q1) | 71 | 2.4% |  | Fertility and Sterility  (7.4, Q1) | 71 | 2332 |  | BMC Medicine  (11.1, Q1) | 7 | 95.2 |
| 5 | Frontiers in Endocrinology  (6.0, Q1) | 71 | 2.4% |  | PloS One  (3.7, Q2) | 56 | 2076 |  | Endocrinology and Metabolism Clinics of North America  (4.7, Q2) | 5 | 78.4 |
| 6 | Clinical Endocrinology  (3.5, Q3) | 66 | 2.3% |  | Gynecological Endocrinology (2.2, Q3) | 156 | 2000 |  | Diabetes  (9.3, Q1) | 7 | 60.4 |
| 7 | PloS One  (3.7, Q2) | 56 | 1.9% |  | Endocrine Reviews  (25.2, Q1) | 6 | 1789 |  | Steroids  (2.7, Q4) | 18 | 51.4 |
| 8 | Nutrients  (6.7, Q1) | 37 | 1.3% |  | Clinical Endocrinology  (3.5, Q3) | 66 | 1496 |  | Cochrane Database of Systematic Reviews  (12.0, Q1) | 6 | 48.3 |
| 9 | Journal of Endocrinological Investigation  (5.4, Q2) | 36 | 1.2% |  | European Journal of Endocrinology  (6.5, Q1) | 28 | 1146 |  | Obesity Reviews  (10.8, Q1) | 12 | 46.2 |
| 10 | European Journal of Obstetrics & Gynecology and Reproductive Biology  (2.8, Q3) | 34 | 1.1% |  | Steroids  (2.7, Q4) | 18 | 926 |  | Maturitas  (5.1, Q1) | 9 | 46.1 |
|  | | | | | | | | | | | |
